# Comparison of cardiac, hepatic, and renal effects of arginine vasopressin and noradrenaline during porcine fecal peritonitis: a randomized controlled trial

**DOI:** 10.1186/cc7959

**Published:** 2009-07-10

**Authors:** Florian Simon, Ricardo Giudici, Angelika Scheuerle, Michael Gröger, Pierre Asfar, Josef A Vogt, Ulrich Wachter, Franz Ploner, Michael Georgieff, Peter Möller, Régent Laporte, Peter Radermacher, Enrico Calzia, Balázs Hauser

**Affiliations:** 1 Sektion Anästhesiologische Pathophysiologie und Verfahrensentwicklung, Klinik für Anästhesiologie, Universitätsklinikum, Steinhövelstrasse 9, 89075 Ulm, Germany; 2 Abteilung Thorax- und Gefäßchirurgie, Universitätsklinikum, Steinhövelstrasse 9, 89075 Ulm, Germany; 3 Instituto di Anestesiologia e Rianimazione dell'Università degli Studi di Milano, Azienda Ospedaliera, Polo Universitario San Paolo, Via di Rudin 8, 20142 Milan, Italy; 4 Abteilung Pathologie, Universitätsklinikum, Albert-Einstein-Allee 11, 89081 Ulm, Germany; 5 Laboratoire HIFIH, UPRES-EA 3859, IFR 132, Universitè d'Angers, Département de Réanimation Médicale et de Médecine Hyperbare, Centre Hospitalo- Universitaire, 4, rue Larrey, 49933 Angers cedex 9, France; 6 Abteilung für Anästhesiologie und Schmerztherapie, Landeskrankenhaus Sterzing, Margarethenstraße 24, 39049 Sterzing, Italy; 7 Ferring Research Institute Inc., 3550 General Atomics Court, Bldg 2 Room 444, San Diego, CA 92121, USA; 8 Semmelweis Egyetem, Aneszteziológiai és Intenzív Terápiás Klinika, Kútvölgyi út 4., 1125 Budapest, Hungary

## Abstract

**Introduction:**

Infusing arginine vasopressin (AVP) in vasodilatory shock usually decreases cardiac output and thus systemic oxygen transport. It is still a matter of debate whether this vasoconstriction impedes visceral organ blood flow and thereby causes organ dysfunction and injury. Therefore, we tested the hypothesis whether low-dose AVP is safe with respect to liver, kidney, and heart function and organ injury during resuscitated septic shock.

**Methods:**

After intraperitoneal inoculation of autologous feces, 24 anesthetized, mechanically ventilated, and instrumented pigs were randomly assigned to noradrenaline alone (increments of 0.05 μg/kg/min until maximal heart rate of 160 beats/min; n = 12) or AVP (1 to 5 ng/kg/min; supplemented by noradrenaline if the maximal AVP dosage failed to maintain mean blood pressure; n = 12) to treat sepsis-associated hypotension. Parameters of systemic and regional hemodynamics (ultrasound flow probes on the portal vein and hepatic artery), oxygen transport, metabolism (endogenous glucose production and whole body glucose oxidation derived from blood glucose isotope and expiratory ^13^CO_2_/^12^CO_2 _enrichment during 1,2,3,4,5,6-^13^C_6_-glucose infusion), visceral organ function (blood transaminase activities, bilirubin and creatinine concentrations, creatinine clearance, fractional Na^+ ^excretion), nitric oxide (exhaled NO and blood nitrate + nitrite levels) and cytokine production (interleukin-6 and tumor necrosis factor-α blood levels), and myocardial function (left ventricular dp/dt_max _and dp/dt_min_) and injury (troponin I blood levels) were measured before and 12, 18, and 24 hours after peritonitis induction. Immediate post mortem liver and kidney biopsies were analysed for histomorphology (hematoxylin eosin staining) and apoptosis (TUNEL staining).

**Results:**

AVP decreased heart rate and cardiac output without otherwise affecting heart function and significantly decreased troponin I blood levels. AVP increased the rate of direct, aerobic glucose oxidation and reduced hyperlactatemia, which coincided with less severe kidney dysfunction and liver injury, attenuated systemic inflammation, and decreased kidney tubular apoptosis.

**Conclusions:**

During well-resuscitated septic shock low-dose AVP appears to be safe with respect to myocardial function and heart injury and reduces kidney and liver damage. It remains to be elucidated whether this is due to the treatment *per se *and/or to the decreased exogenous catecholamine requirements.

## Introduction

Infusing arginine vasopressin (AVP) in vasodilatory septic shock is usually accompanied by a decrease in cardiac output and systemic oxygen (O_2_) transport. It is still a matter of debate whether this vasoconstriction impedes visceral organ blood flow and thereby causes organ dysfunction [1-5]. In fact, controversial data have been reported in experimental [6-19] and clinical studies [20-22]. The vasopressin-induced vasoconstriction is also associated with reduced coronary flow, but again data are equivocal [23-27], most likely because of the variable impact of coronary flow and perfusion pressure [27]. Consequently, the use of vasopressin is still cautioned in patients with heart and/or peripheral vascular disease [2,3,5], and the multicenter Vasopressin and Septic Shock Trial (VASST) explicitly excluded patients with cardiogenic shock, ischemic heart disease, congestive heart failure, and mesenteric ischemia [27].

Given this controversy, we tested the hypothesis whether low-dose AVP infusion (supplemented with noradrenaline) is safe with respect to liver, kidney, and heart function in a clinically relevant porcine model of fecal peritonitis-induced septic shock [28]. AVP was compared with noradrenaline, and the two drugs were titrated to maintain comparable blood pressure.

## Materials and methods

### Animal preparation, measurements, and calculations

The study protocol was approved by the University Animal Care Committee and the Federal Authorities for Animal Research (Regierungspräsidium Tübingen, Germany, Reg.-Nr III/15). Anesthesia, surgical instrumentation, measurements have been described in detail previously [28]. Systemic, pulmonary, and hepatic (ultrasound flow probes on the portal vein and the hepatic artery) hemodynamics and gas exchange (calorimetric O_2 _uptake and carbon dioxide (CO_2_) production, arterial, portal, hepatic, and mixed venous blood gases and oximetry), intrathoracic blood volume, extravascular lung water and indocyanine-green plasma disappearance rate (thermal-green dye double indicator dilution), blood glucose, lactate, pyruvate, bilirubin, creatinine, troponin I, nitrate+nitrite (NO_2_^-^+NO_3_^-^; chemoluminescence), TNFα, and IL-6 concentrations, as well as the alanine aminotransferase (ALAT) and aspartate aminotransferase (ASAT) activities were determined as described previously [28]. The bilirubin, creatinine, troponin I, IL-6, TNF-α and NO_2_^-^+NO_3_^- ^concentrations and the ALAT and ASAT activities are normalized per gram of plasma protein to correct for dilution by intravenous fluids [28]. Endogenous glucose production and direct, aerobic glucose oxidation were derived from the rate of appearance of stable, non-radioactively labeled 1,2,3,4,5,6-^13^C_6_-glucose and the mixed expiratory ^13^CO_2_, respectively, during continuous intravenous isotope infusion, after gas chromatography-mass spectrometry assessment of plasma and non-dispersive infrared spectrometry measurement of expiratory gas isotope enrichment [28]. Left ventricular function was evaluated using a pressure tip catheter (Millar Mikro-Tip^®^, Millar Instruments, Houston, TX, USA) that allowed measuring maximal systolic contraction (dp/dt_max_) and diastolic relaxation (dp/dt_min_), as well as the frequency-independent relaxation time (τ).

Immediate postmortem liver, kidney, and heart biopsies were evaluated for histomorphologic changes (H&E staining) and the number of apoptotic nuclei (terminal deoxynucleotidyltransferase-mediated nick-end labeling-assay (TUNEL) staining) [28]. Evidence of apoptosis was accepted only if nuclear staining was considered TUNEL positive, the scores reported representing the number of positive nuclear stainings. Slides were evaluated by a pathologist (AS) blinded for the group assignment.

### Experimental protocol

Body temperature was kept between 37 and 39°C, that is ± 1°C of the pre-peritonitis value, with heating pads or cooling. Ventilator settings were [28]: tidal volume 8 mL/kg, positive end expiratory pressure (PEEP) 10 cmH_2_O, inspiratory-to-expiratory (I/E) ratio 1:1.5, respiratory rate adjusted to partial pressure of arterial carbon dioxide (PaCO_2_) 35 to 45 mmHg (but maximum 40 mmHg/min), peak airway pressure less than 40 cmH_2_O, fraction of inspired oxygen (FiO_2_) 0.3 (thereafter adjusted to maintain arterial hemoglobin O_2 _saturation > 90%). If partial pressure of arterial oxygen (PaO_2_)/FiO_2 _less than 300 mmHg or less than 200 mmHg, I/E ratio was increased to 1:1 and PEEP to 12 or 15 cmH_2_O, respectively. Lactated Ringer's solution was infused as maintenance fluid (7.5 mL/kg/h), and normoglycemia (4 to 6 mmol/L) was achieved with continuous intravenous glucose as needed. Following instrumentation, an eight-hour recovery period, and baseline data collection, peritonitis was induced by intraperitoneal instillation of 1.0 g/kg autologous feces incubated in 100 mL 0.9% saline for 12 hours at 38°C [28]. Hydroxyethyl-starch (15 mL/kg/h, 10 mL/kg/h if central venous or pulmonary artery occlusion pressure more than 18 mmHg and titrated to maintain intrathoracic blood volume at 25 to 30 mL/kg [28]) allowed the maintainence of a hyperdynamic circulation. When mean blood pressure fell by more than 10% below the pre-peritonitis levels over more than 15 minutes, animals randomly received either noradrenaline (controls: n = 12, 4 males, 8 females, body weight 47 kg, range 38 to 61 kg), titrated in increments of 0.05 μg/kg/min every five minutes until the pre-peritonitis values was reached, or AVP (n = 12, 5 males, 7 females, body weight 46 kg, range 36 to 54 kg), titrated in increments of 1 ng/kg/min every 30 minutes. According to our previous experience [28] we aimed to maintain the pre-peritonitis blood pressure, because, to the best of our knowledge, no data are available on the blood pressure necessary to maintain visceral organ perfusion in septic swine. To avoid tachycardia-induced myocardial ischemia the noradrenaline infusion rate was not further increased if heart rate was 160 beats/min or above. The AVP dose was limited to a maximum infusion rate of 5 ng/kg/min and supplemented by noradrenaline if it failed to maintain blood pressure alone. After additional data collection at 12, 18, and 24 hours of peritonitis, animals were euthanized under deep anesthesia.

### Statistical analysis

Data are presented as median (quartiles) unless otherwise stated. After exclusion of normal distribution using the Kolmogorov-Smirnoff-test, differences within groups were analyzed using a Friedmann analysis of variance on ranks and a subsequent Dunn's test with Bonferroni correction. As our primary hypothesis had been that AVP was safe with respect to liver and heart function in our model, intergroup differences for blood ASAT and ALAT activities as well as bilirubin and troponin I levels were tested using a Mann-Whitney rank sum test with Bonferroni adjustment for multiple comparisons. Because of the multiple statistical testing of the numerous variables measured, all other intergroup comparisons have to be interpreted in a secondary, exploratory, and hypotheses-generating, rather than confirmatory, manner.

## Results

One animal in the control group died following data collection at 18 hours, and thus statistical analysis at 24 hours comprises 23 animals. Colloid resuscitation was identical in the two groups (controls: 15 (14 to 15), AVP: 14 (13 to 14) mL/kg/h). AVP-treated animals did not require any additional noradrenaline during the first 12 hours of the experiment, and, consequently, the median duration and rate of the noradrenaline infusion were significantly lower (duration: 111 (0 to 282) versus 752 (531 to 935) minutes; infusion rate: 0.06 (0.00 to 0.10) versus 0.61 (0.33 to 0.72) μg/kg/min).

**Figure 1:**
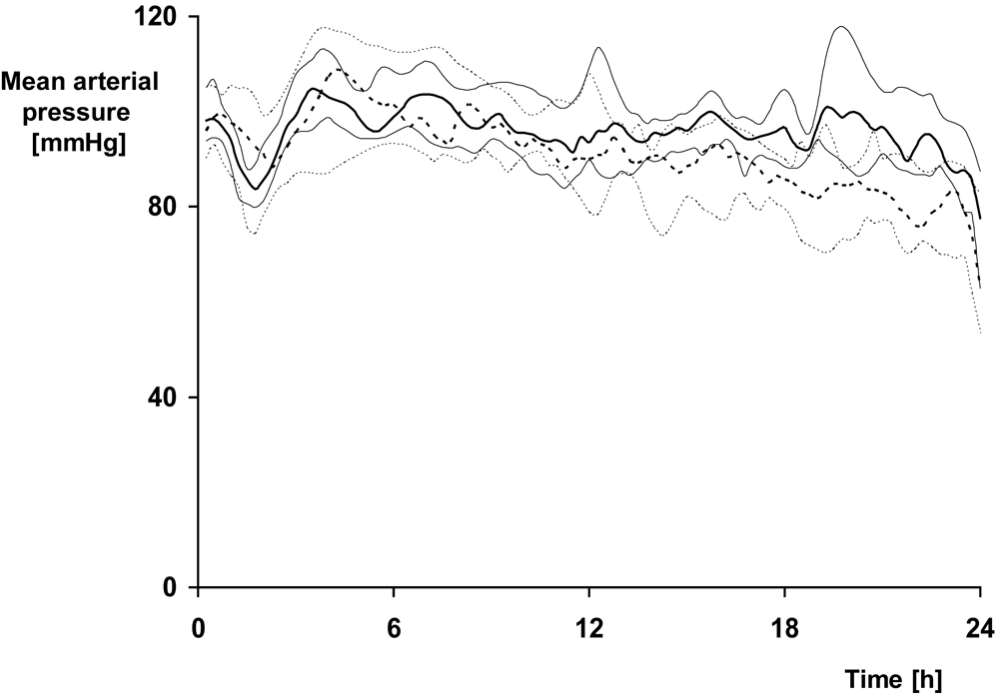
Mean blood pressure in the control and AVP animals. Control = dotted line; n = 12, n = 11 from 20 to 24 hours. Arginine vasopressin (AVP) animals = straight line; n = 12. Data are median (quartiles) and represent a minute-to-minute average based on continuous recording.

**Figure 2:**
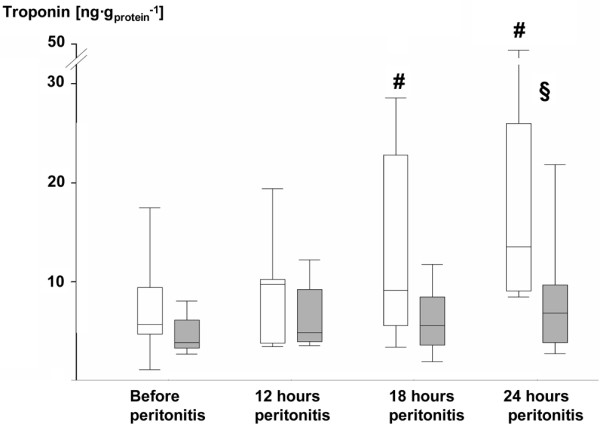
Blood troponin I levels in the control and AVP animals. control = open whiskers; n = 12, n = 11 at 24 hours. Arginine vasopressin (AVP) animals = grey whiskers; n = 12. Data are median (quartiles, range). **# ***P *< 0.05 within groups versus before peritonitis; **§ ***P *< 0.05 between norepinephrine- and AVP-treated animals.

Tables 1 and 2 and Figures 1 and 2 summarize the data on systemic hemodynamics and left heart function (Table 1), as well as O_2 _exchange, acid-base status, and metabolism (Table 2). AVP-treated animals presented with significantly lower heart rate and cardiac output. In contrast to the AVP group, maintenance of mean blood pressure was only achieved in one-third of the control animals, because the noradrenaline infusion rates were not further increased if tachycardia more than 160 beats/min occurred. Nevertheless, albeit mean blood pressure was significantly lower at 18 and 24 hours of peritonitis, one control animal only developed hypotension with a mean blood pressure less than 60 mmHg (Figure 1). None of the other parameters of systemic and pulmonary hemodynamics showed any significant intergroup difference. Although dp/dt_max _was significantly lower in the AVP-treated animals, dp/dt_min _and the diastolic relaxation time τ were comparable in the two groups. Troponin I levels progressively increased in the control animals and were significantly higher than in the AVP group at the end of the experiment (Figure 2). Control animals showed a significantly higher systemic O_2 _transport as well as O_2 _uptake and CO_2 _production, whereas arterial blood gas tensions were nearly identical. The progressive fall of arterial pH and base excess was attenuated in the AVP-treated group (*P *= 0.069 and *P *= 0.053, respectively, at 24 hours). Although the rate of whole body glucose oxidation increased comparably, the progressive rise of endogenous glucose production rate was less pronounced in the AVP animals (*P *= 0.053, *P *= 0.061, and *P *= 0.053 at 12, 18, and 24 hours of peritonitis). Consequently, the directly oxidized fraction of the glucose released was significantly higher in the AVP group, which coincided with significantly lower arterial lactate levels at 18 and 24 hours.

**Table 1:** Parameters of systemic hemodynamics and cardiac function in the control (n = 12, n = 11 at 24 hours of peritonitis) and AVP (n = 12) groups

		Before peritonitis	12 hours peritonitis	18 hours peritonitis	24 hours peritonitis
Heart rate	Control	92 (87 to 104)	128 (105 to 153)^b^	155 (129 to 160)^b^	158 (154 to 160)^b^
(beats/min)	AVP	85 (75 to 95)	96 (76 to 102)^a^	87 (74 to 105)^a^	103 (84 to 112)^a, b^
Cardiac output	Control	105 (95 to 119)	122 (101 to 129)	155 (125 to 167)^b^	131 (117 to 183)^b^
(mL/kg/min)	AVP	105 (95 to 107)	95 (84 to 105)	97 (71 to 122)^a^	104 (82 to 136)
Mean arterial	Control	98 (93 to 105)	95 (82 to 108)	89 (72 to 91)^b^	78 (63 to 89)^b^
pressure (mmHg)	AVP	95 (90 to 104)	96 (90 to 111)	99 (91 to 104)^a^	98 (90 to 102)^a^
Mean pulmonary artery	Control	27 (26 to 30)	37 (34 to 42)^b^	36 (32 to 41)^b^	39 (34 to 44)^b^
pressure (mmHg)	AVP	28 (26 to 30)	37 (31 to 43)^b^	37 (36 to 40)^b^	40 (37 to 44)^b^
Central venous	Control	12 (12 to 14)	14 (12 to 16)	15 (13 to 18)^b^	19 (14 to 21)^b^
pressure (mmHg)	AVP	12 (12 to 13)	16 (14 to 17)^b^	16 (14 to 17)^b^	17 (16 to 19)^b^
Pulmonary artery occlusion	Control	14 (13 to 16)	16 (14 to 17)	16 (13 to 18)	17 (14 to 19)^b^
pressure (mmHg)	AVP	13 (12 to 15)	16 (13 to 16)	17 (15 to 18)^b^	18 (18 to 19)^b^
Stroke volume	Control	1.2 (11 to 1.4)	0.9 (0.9 to 1.0)^b^	1.0 (0.9 to 1.1)	0.9 (0.8 to 1.2)
(mL/kg)	AVP	1.2 (1.0 to 1.3)	1.0 (0.9 to 1.3)^b^	1.0 (0.9 to 1.2)	1.0 (0.9 to 1.1)
Intrathoracic blood volume	Control	27 (22 to 35)	25 (23 to 26)	28 (26 to 31)	27 (26 to 32)
(mL/kg)	AVP	26 (21 to 29)	24 (21 to 28)	29 (24 to 31)	21 (20 to 28)
DP/dt_max_	Control	1355 (1246 to 1415)	1774 (1663 to 1980)	2011 (1291 to 2215)	1532 (1119 to 1979)
(mmHg/sec)	AVP	1137 (957 to 1410)	793 (758 to 844)^a^	893 (739 to 1310)	915 (730 to 1404)^a^
DP/dt_min_	Control	-1296 (-1329 to -1134)	-1444 (-1556 to -1093)	-1421 (-1709 to -948)	-1243 (-1493 to -1038)
(mmHg/sec)	AVP	-1321 (-1476 to -1128)	-1065 (-1114 to -890)	-1202 (-1311 to -930)	-1109 (-1473 to -887)^b^
τ	Control	22 (20 to 22)	25 (17 to 26)	23 (18 to 26)	20 (18 to 25)
(ms)	AVP	22 (20 to 25)	19 (15 to 20)	21 (16 to 23)	19 (15 to 25)^b^

All data are median (quartiles). ^a ^*P *< 0.05 between norepinephrine- and AVP-treated animals; ^b ^*P *< 0.05 within groups versus before peritonitis.

AVP = arginine vasopressin; dp/dt_max _= maximal systolic contraction; dp/dt_min _= maximal diastolic relaxation.

**Table 2:** Parameters of systemic gas exchange, metabolism and acid-base status in the control (n = 12, n = 11 at 24 hours of peritonitis) and AVP (n = 12) groups

		Before peritonitis	12 hours peritonitis	18 hours peritonitis	24 hours peritonitis
Arterial PO_2_	Control	166 (160 to 179)	144 (124 to 153)^b^	106 (93 to 121)^b^	87 (80 to 114)^b^
(mmHg)	AVP	163 (154 to 179)	144 (128 to 170)^b^	124 (96 to 150)^b^	96 (84 to 138)^b^
Arterial PCO_2_	Control	37 (35 to 39)	41 (40 to 44)^b^	41 (39 to 45)^b^	44 (39 to 46)^b^
(mmHg)	AVP	36 (34 to 40)	40 (39 to 43)^b^	41 (38 to 44)^b^	42 (39 to 45)^b^
Extravascular lung water	Control	4.4 (3.0 to 6.0)	4.8 (1.5 to 7.0)	5.8 (1.4 to 8.6)	7.4 (5.5 to 8.6)^b^
(mL/kg)	AVP	3.3 (2.7 to 5.0)	7.4 (1.8 to 9.6)^b^	9.0 (1.1 to 11.0)^b^	5.9 (3.4 to 8.4)^b^
Systemic O_2 _delivery	Control	10 (9 to 11)	14 (11 to 18)^b^	19 (16 to 23)^b^	17 (12 to 21)^b^
(mL/kg/min)	AVP	11 (10 to 12)	11 (11 to 13)	12 (8 to 15)^a^	13 (10 to 16)
Systemic O_2 _uptake	Control	4.9 (4.0 to 5.3)	4.4 (3.7 to 5.7)	6.0 (4.5 to 7.2)^b^	6.0 (5.3 to 6.8)^b^
(mL/kg/min)	AVP	4.7 (4.2 to 4.8)	4.6 (3.9 to 4.7)^b^	4.7 (4.2 to 4.9)^a^	4.7 (4.2 to 5.6)^a^
Systemic CO_2 _production	Control	3.1 (2.7 to 3.5)	3.5 (3.0 to 4.1)^b^	4.1 (3.7 to 4.5)^b^	4.4 (4.0 to 4.8)^b^
(mL/kg/min)	AVP	3.0 (2.7 to 3.4)	3.2 (2.9 to 3.6)	3.4 (3.1 to 3.6)^a, b^	3.5 (3.2 to 3.8)^a, b^
Endogenous glucose	Control	2.7 (2.4 to 3.4)	5.6 (4.5 to 6.3)^b^	7.2 (5.6 to 8.4)^b^	7.7 (7.1 to 10.2)^b^
production (mg/kg/min)	AVP	2.5 (2.2 to 2.9)	4.5 (4.0 to 4.8)^b^	4.9 (4.7 to 6.8)^b^	6.6 (5.0 to 7.5)^b^
Systemic glucose	Control	1.9 (1.4 to 2.9)	3.2 (2.1 to 3.4)^b^	3.8 (3.1 to 4.3)^b^	3.8 (3.4 to 4.5)^b^
oxidation (mg/kg/min)	AVP	1.9 (1.6 to 2.4)	2.9 (2.5 to 3.8)^b^	3.7 (2.9 to 3.9)^b^	3.8 (3.2 to 4.2)^b^
Glucose oxidation/production ratio (%)	Control	74 (50 to 104)	54 (51 to 62)^b^	52 (50 to 56)	49 (44 to 55)^b^
	AVP	79 (60 to 93)	64 (57 to 72)^a^	62 (57 to 64)^a, b^	57 (53 to 65)^a, b^
Arterial lactate	Control	0.9 (0.8 to 1.0)	1.1 (1.0 to 1.3)^b^	2.0 (1.3 to 3.6)^b^	2.3 (1.8 to 4.1)^b^
(mmol/L)	AVP	0.9 (0.8 to 1.0)	0.9 (0.8 to 1.1)	1.2 (1.0 to 1.5)^a, b^	1.5 (1.3 to 1.9)^a, b^
Arterial	Control	8 (7 to 9)	12 (11 to 13)	13 (12 to 16)^a^	15 (13 to 17)^a^
lactate/pyruvate ratio	AVP	9 (8 to 10)	12 (11 to 13)	12 (11 to 13)^a^	14 (13 to 15)^a^
Arterial pH	Control	7.56 (7.55 to 7.59)	7.50 (7.45 to 7.53)^b^	7.47 (7.44 to 7.49)^b^	7.44 (7.38 to 7.45)^b^
	AVP	7.54 (7.49 to 7.57)	7.51 (7.49 to 7.52)^b^	7.49 (7.45 to 7.53)^b^	7.49 (7.44 to 7.51)^b^
Arterial base excess	Control	10.3 (8.8 to 12.3)	9.9 (7.0 to 11.3)	6.0 (3.4 to 8.0)^b^	4.1 (-0.2 to 6.2)^b^
(mmol/L)	AVP	9.3 (7.9 to 11.0)	9.6 (8.3 to 11.1)	8.9 (6.1 to 9.4)	7.1 (3.9 to 10.7)

All data are median (quartiles). ^a ^*P *< 0.05 between norepinephrine- and AVP-treated animals; ^b ^*P *< 0.05 within groups versus before peritonitis.

AVP = arginine vasopressin; PCO_2 _= partial pressure of carbon dioxide; PO_2 _= partial pressure of oxygen.

**Figure 3:**
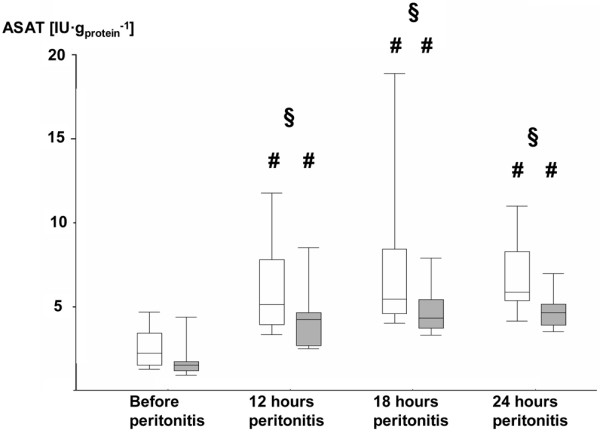
Blood ASAT activities as levels in the control and AVP animals. Control = open whiskers; n = 12, n = 11 at 24 hours. Arginine vasopressin (AVP) animals = grey whiskers, n = 12. Data are median (quartiles, range). **# ***P *< 0.05 within groups versus before peritonitis; ***§ ****P *< 0.05 between norepinephrine- and AVP-treated animals. ASAT = asparatate aminotransferase.

**Figure 4:**
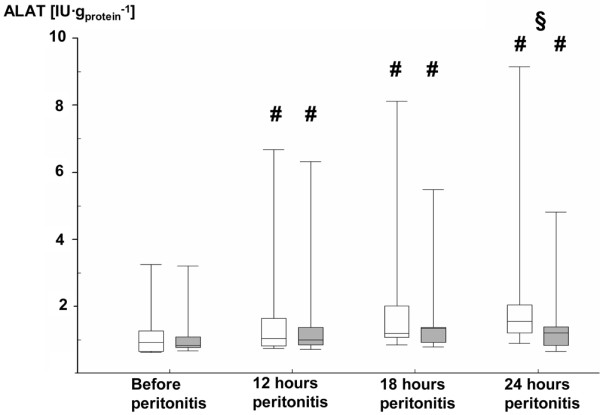
Blood ALAT levels in the control and AVP animals. Control = open whiskers; n = 12, n = 11 at 24 hours. Arginine vasopressin (AVP) animals = grey whiskers; n = 12. Data are median (quartiles, range). **# ***P *< 0.05 within groups versus before peritonitis; ***§ ****P *< 0.05 between norepinephrine- and AVP-treated animals. ALAT = alanine aminotransferase.

**Figure 5:**
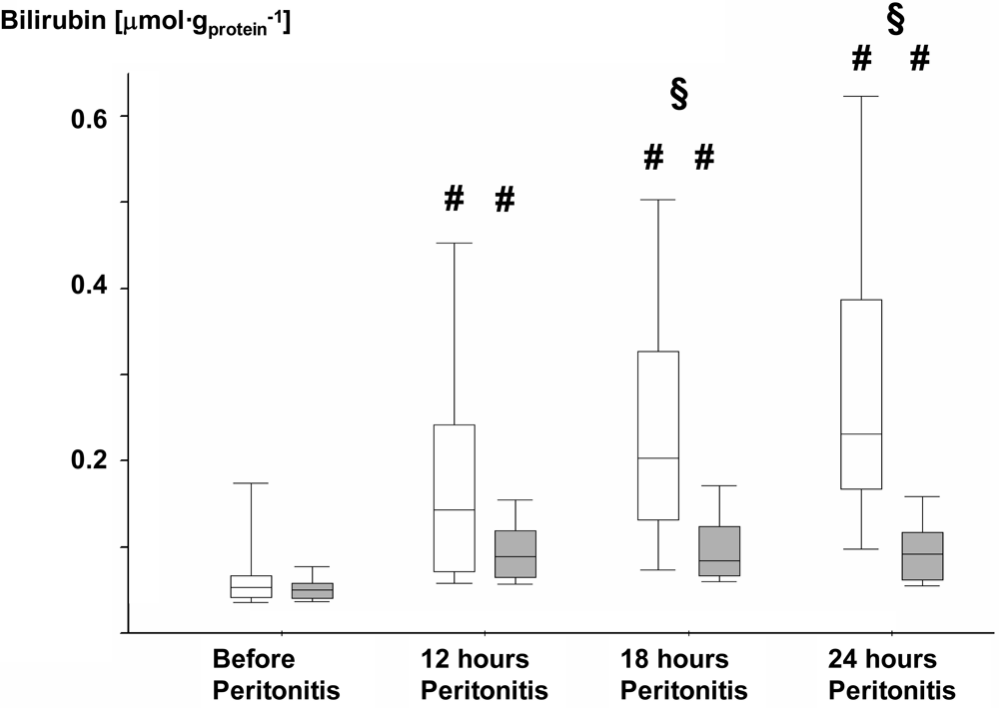
Blood bilirubin levels in the control and AVP animals. Control = open whiskers; n = 12, n = 11 at 24 hours. Arginine vasopressin (AVP) animals = grey whiskers; n = 12. Data are median (quartiles, range). **# ***P *< 0.05 within groups versus before peritonitis; **§ ***P *< 0.05 between norepinephrine- and AVP-treated animals.

**Figure 6:**
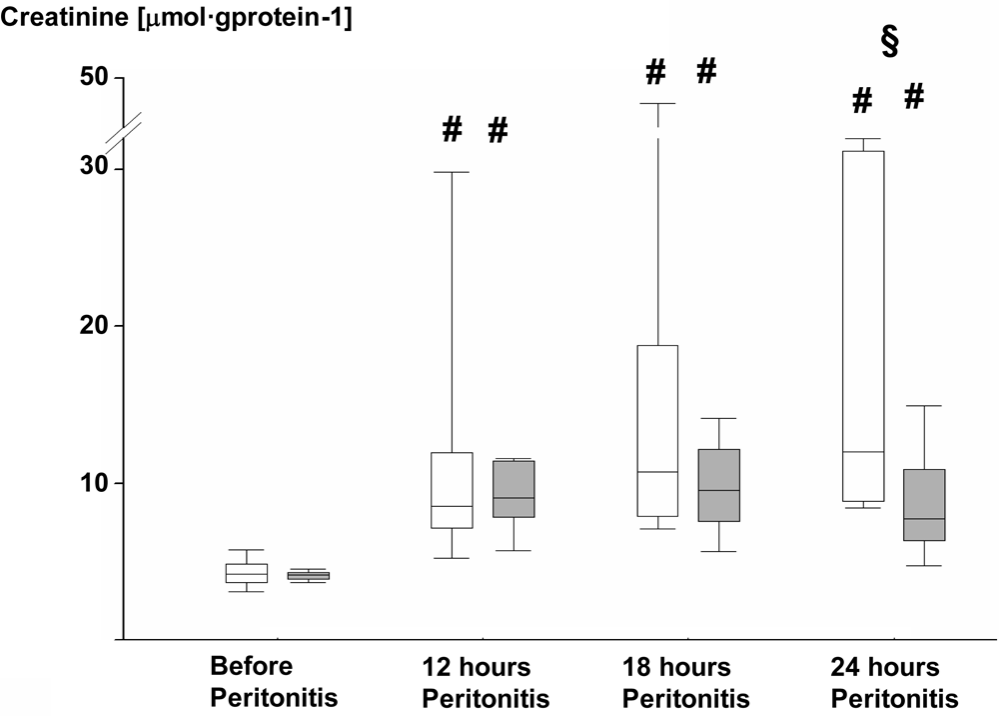
Blood creatinine levels in the control and AVP animals. Control = open whiskers; n = 12, n = 11 at 24 hours. Arginine vasopressin (AVP) animals = grey whiskers; n = 12. Data are median (quartiles, range). **# ***P *< 0.05 within groups versus before peritonitis; **§ ***P *< 0.05 between norepinephrine- and AVP-treated animals.

Table 3 and Figures 3, 4, 5 and 6 summarize the parameters of visceral organ blood flow, O_2 _kinetics, acid-base status, and function. Except for a lower portal venous flow (*P *= 0.053 at 24 hours), liver hemodynamics and O_2 _exchange did not significantly differ between the two groups. Nevertheless, AVP attenuated the portal and hepatic venous acidosis (Table 3) and blunted the otherwise significant rise in serum transaminase activities and bilirubin levels (Figures 3, 4 and 5). AVP prevented the time-dependent fall in urine output so that diuresis was significantly higher between 12 and 24 hours (Table 3). Renal dysfunction with reduced creatinine clearance (Table 3) and increased blood creatinine levels (Figure 6) was less severe, while fractional Na^+ ^excretion was significantly higher in the AVP-treated animals (Table 3).

**Table 3:** Parameters of visceral organ (liver, kidney) hemodynamics, acid-base status and organ function in the control (n = 12, n = 11 at 24 hours of peritonitis) and AVP (n = 12) groups

		Before peritonitis	12 hours peritonitis	18 hours peritonitis	24 hours peritonitis
Portal vein flow (mL/kg/min)	Control	18 (15 to 22)	29 (21 to 31)^b^	29 (24 to 34)^b^	26 (24 to 30)^b^
	AVP	18 (16 to 20)	24 (20 to 31)^b^	22 (16 to 27)	20 (16 to 24)
Hepatic artery flow (mL/kg/min)	Control	1.7 (0.4 to 2.1)	1.4 (0.9 to 2.9)	1.6 (1.3 to 3.5)	2.1 (1.1 to 3.6)^b^
	AVP	0.6 (0.2 to 1.6)	1.6 (0.2 to 3.2)^b^	1.9 (0.3 to 3.3)^b^	3.0 (0.3 to 5.5)^b^
Hepatic O_2 _delivery (mL/kg/min)	Control	1.0 (0.9 to 1.5)	2.9 (2.5 to 3.7)^b^	3.0 (2.0 to 3.5)^b^	2.6 (1.8 to 3.1)^b^
	AVP	1.2 (1.0 to 1.5)	2.5 (1.9 to 3.0)^b^	2.2 (1.7 to 3.0)^b^	2.3 (1.4 to 2.7)^b^
Portal vein O_2 _saturation (%)	Control	58 (55 to 64)	78 (76 to 81)^b^	77 (71 to 79)^b^	72 (67 to 74)^b^
	AVP	60 (55 to 63)	78 (68 to 83)^b^	72 (65 to 75)^b^	69 (63 to 71)^b^
Hepatic vein O_2 _saturation (%)	Control AVP	25 (24 to 72)	63 (54 to 65)^b^	58 (52 to 65)^b^	53 (44 to 56)^b^
		30 (20 to 55)	66 (50 to 70)^b^	54 (42 to 61)^b^	55 (50 to 58)^b^
Portal drained viscera O_2 _extraction (%)	Control AVP	40 (37 to 46)	21 (18 to 24)^b^	21 (18 to 25)^b^	27 (24 to 34)^b^
		43 (37 to 44)	22 (17 to 35)^b^	22 (19 to 31)^b^	30 (25 to 34)^b^
Hepatic O_2 _uptake	Control	0.6 (0.4 to 0.8)	0.6 (0.4 to 0.9)	0.7 (0.5 to 1.1)	0.6 (0.4 to 0.8)
(mL/kg/min)	AVP	0.6 (0.5 to 0.9)	0.8 (0.5 to 0.9)	0.7 (0.4 to 1.0)	0.5 (0.3 to 0.7)
Portal vein	Control	10 (9 to 12)	14 (12 to 15)	15 (13 to 17)	16 (13 to 18)^a^
lactate/pruvate ratio	AVP	11 (10 to 12)	13 (11 to 15)	14 (13 to 15)	15 (13 to 17)^a^
Hepatic vein	Control	9 (8 to 10)	12 (10 to 15)	13 (12 to 15)	14 (12 to 18)^a^
lactate/pruvate ratio	AVP	8 (7 to 12)	12 (10 to 15)	11 (10 to 16)	13 (11 to 16)^a^
Portal vein pH	Control	7.49 (7.46 to 7.52)	7.46 (7.42 to 7.48)	7.41 (7.38 to 7.45)^b^	7.37 (7.33 to 7.42)^b^
	AVP	7.48 (7.43 to 7.51)	7.47 (7.44 to 7.49)^b^	7.44 (7.39 to 7.47)^b^	7.42 (7.37 to 7.43)^b^
Hepatic vein pH	Control	7.49 (7.47 to 7.53)	7.48 (7.43 to 7.49)	7.43 (7.40 to 7.46)^b^	7.39 (7.33 to 7.44)^b^
	AVP	7.49 (7.44 to 7.54)	7.47 (7.44 to 7.50)	7.43 (7.39 to 7.48)^b^	7.44 (7.40 to 7.46)
Portal vein base excess (mmol/L)	Control	10.8 (9.5 to 12.5)	10.2 (8.1 to 11.2)^b^	6.5 (3.0 to 8.2)^b^	4.8 (0.1 to 6.2)^b^
	AVP	9.8 (7.8 to 12.4)	9.2 (7.3 to 10.4)	9.5 (6.0 to 10.6)	8.9 (3.0 to 11.0)^a^
Hepatic vein base excess (mmol/L)	Control	12.6 (10.5 to 14.2)	11.1 (7.9 to 12.2)^b^	7.6 (5.1 to 8.9)^b^	5.8 (0.5 to 7.4)^b^
	AVP	11.6 (10.1 to 14.8)	10.5 (8.5 to 12.2)^b^	9.8 (4.5 to 11.1)^b^	9.0 (3.8 to 11.8)^b^
ICG plasma	Control	20 (19 to 23)	17 (13 to 31)	14 (10 to 34)	13 (8 to 22)^b^
disappearance rate (%/min)	AVP	15 (11 to 19)	14 (10 to 18)	13 (8 to 15)	12 (12 to 15)
Urine output (mL/kg/h)	Control	5.4 (4.1 to 7.2)		3.2 (2.3 to 4.8)^b^	
	AVP	6.7 (5.9 to 8.0)		5.6 (4.6 to 8.6)^a^	
Creatinine clearance (mL/min)	Control	80 (67 to 88)		64 (35 to 85)^c^	
	AVP	79 (60 to 98)		61 (44 to 73)^c^	
Fractional Na^+ ^excretion (%)	Control AVP	5.6 (4.8 to 7.7)		3.0 (2.5 to 5.1)	
		8.3 (6.4 to 10.0)^a^		9.5 (7.2 to 10.7)^a^	

Data on urine flow, creatinine clearance, and fractional Na^+ ^excretion refer to the first and second half of the experiment, respectively. All data are median (quartiles). ^a ^*P *< 0.05 between norepinephrine- and AVP-treated animals; ^b ^*P *< 0.05 within groups versus before peritonitis.

AVP = arginine vasopressin; ICG = indocyanine-green dye.

Table 4 shows the parameters of the inflammatory response. Although the increase in blood NO_2_^-^+NO_3_^- ^and TNFα levels was comparable, AVP was associated with significantly lower IL-6 concentrations and expired nitric oxide (NO).

**Table 4:** Parameters of systemic NO and cytokine production in the control (n = 12, n = 11 at 24 hours of peritonitis) and AVP (n = 12) groups

		Before peritonitis	12 hours peritonitis	18 hours peritonitis	24 hours peritonitis
Exhaled NO (pmol/kg/min)	Control	6 (3 to 47)	22 (6 to 72)^b^	27 (11 to 98)^b^	15 (14 to 141)^b^
	AVP	5 (4 to 9)	14 (7 to 17)^b^	12 (9 to 16)^b^	8 (6 to 10)^a^
Arterial NO_3_^-^+NO_2_^- ^(μmol/g_protein_)	Control	0.5 (0.4 to 1.6)	1.5 (0.6 to 2.1)^b^	1.8 (0.9 to 2.6)^b^	1.8 (1.3 to 2.7)^b^
	AVP	1.0 (0.6 to 1.3)	1.4 (1.0 to 2.2)^b^	1.3 (1.0 to 2.4)^b^	1.2 (1.0 to 2.3)^b^
Tumor necrosis factor-α (μmol/g_protein_)	Control	3 (2 to 3)	10 (8 to 16)^b^	20 (12 to 25)^b^	27 (15 to 55)^b^
	AVP	2 (2 to 3)	8 (7 to 11)^b^	14 (12 to 19)^b^	18 (15 to 29)^b^
Interleukin 6 (μmol/g_protein_)	Control	1 (1 to 1)	125 (56 to 286)^b^	549 (252 to 1624)^b^	753 (559 to 3443)^b^
	AVP	1 (0 to 3)	83 (51 to 150)^b^	216 (119 to 365)^a, b^	354 (140 to 677)^a, b^

All data are median (quartiles). ^a ^*P *< 0.05 between norepinephrine- and AVP-treated animals; ^b ^*P *< 0.05 within groups versus before peritonitis. AVP = arginine vasopressin; NO = nitric oxide.

Histomorphologic evaluation showed some non-specific subcapsular inflammatory cell infiltration and a few biliary tract concrements in the liver, and tubular swelling in the kidney; however, this was without any intergroup difference, and no pathologic findings at all in the myocardium. Although TUNEL-positive nuclei were absent or rare (without intergroup difference) in the heart and the liver, respectively, AVP-treated animals showed less TUNEL-positive renal tubular nuclei (3 (3 to 9) versus 11 (5 to 15), respectively, *P *= 0.061).

## Discussion

The aim of the present study was to test the hypothesis whether low-dose AVP infusion is safe for heart and visceral organ function in a clinically relevant, resuscitated, and hyperdynamic porcine model of fecal peritonitis-induced septic shock. AVP supplemented with noradrenaline was compared with noradrenaline alone, which were titrated to maintain comparable blood pressure. The key findings were that: AVP decreased heart rate and cardiac output without affecting myocardial relaxation, and significantly decreased troponin I blood levels; increased the rate of direct, aerobic glucose oxidation, and reduced hyperlactatemia; attenuated kidney dysfunction as well as liver injury, which coincided with less severe systemic inflammatory response.

In our experiment, left ventricular dp/dt_max _was significantly lower in the AVP group, whereas dp/dt_min _remained unchanged. Thus our experiment seems to confirm negative inotrope properties of AVP in isolated hearts [23,24] and endotoxin-challenged rabbits [25]. As first derivatives of pressure, dp/dt_max _and dp/dt_min _crucially depend on heart rate. In the mentioned studies, however, heart rate was not affected at all [23,24] or decreased by less than 10% only [25]. Furthermore, an unresuscitated model with endotoxin-induced cardiac dysfunction [25] or AVP decreased coronary blood flow below baseline levels [23,24]. Clearly, as we did not measure coronary blood flow, we cannot exclude a vasoconstriction-related reduction in coronary perfusion. Nevertheless, it is unlikely that AVP caused myocardial ischemia: troponin I levels progressively increased in the control animals only and were significantly higher than in the AVP group at the end of the experiment. Our findings are in sharp contrast to data by Müller and colleagues, who recently reported unchanged systolic and compromised diastolic heart function during incremental AVP infusion in swine with transient myocardial ischemia [18]. These authors also studied a hypodynamic model characterized by a reduced cardiac output resulting from myocardial dysfunction, while we investigated fluid-resuscitated animals with a sustained increase in cardiac output. In addition, Müller and colleagues infused AVP alone, while we combined AVP with noradrenaline. In fact, the current rationale of AVP use comprises a supplemental infusion, targeted to restore vasopressin levels, simultaneously with catecholamines rather than AVP alone [29]. It remains open whether the results reported by Müller and colleagues were due to the AVP-related vasoconstriction, that is, afterload-dependent and/or related to coronary hypoperfusion, or to a genuine myocardial effect. This issue, however, is critical in the discussion on cardiac effects of AVP: 'cardiac efficiency', that is, the product of left ventricular pressure times heart rate normalized for myocardial O_2 _consumption, was well maintained under constant flow conditions [26]. Finally, the significantly reduced noradrenaline requirements may have contributed to the less severe myocardial injury [30]. In the control group, maintaining blood pressure at pre-peritonitis levels necessitated high noradrenaline infusion rates, which were reported to cause myocardial injury due to increased workload [31] and reduced metabolic efficiency resulting from enhanced fatty acid oxidation [32].

Despite the lower portal venous flow infusing AVP did not have any detrimental effect on liver O_2 _exchange and, moreover, was associated with less severe hepatic venous metabolic acidosis and attenuated liver injury. Furthermore, AVP infusion resulted in significantly less severe kidney dysfunction. Controversial effects were reported on the effects of AVP infusion on visceral organ blood flow and function during large animal sepsis and septic shock: although AVP decreased mesenteric arterial and portal venous flow during porcine and ovine bacterial sepsis [13,15,16] or endotoxemia [6,7,10], other studies found unchanged hepato-splanchnic perfusion when vasopressin or terlipressin were infused during hyperdynamic porcine endotoxemia and ovine fecal peritonitis [8,10,19]. The effect of AVP on the kidney macrocirculation was even more heterogenous, in as much decreased [10], unchanged [13,16], and even increased [7] renal blood flow were reported. It should be emphasized that a fall in regional blood flow below baseline levels associated with signs of organ ischemia, for example, regional venous acidosis and/or increased lactate concentrations, only occurred in hypodynamic models with a sustained decrease in cardiac output [7,10] and/or with AVP doses higher than currently recommended [15,16]. In fact, Sun and colleagues demonstrated during ovine fecal peritonitis that both low-dose vasopressin alone and in combination with noradrenaline were associated with less severe hyperlactatemia and tissue acidosis than with noradrenaline alone, which ultimately resulted in improved survival [8]. In endotoxic swine infusing low doses of the AVP analogue terlipressin also caused hyperlactatemia, which, however, did not originate from the hepato-splanchnic system and was even associated with attenuated portal and hepatic venous metabolic acidosis [33].

AVP did not affect creatinine clearance, and fractional Na^+ ^excretion was significantly increased. Therefore, it could be argued that AVP deteriorated or, at best, did not influence kidney function [34], which would be in contrast with previous reports of improved renal function in experimental models [9,13,35] and clinical investigations [22,36]. It should be noted, however, that AVP significantly attenuated the otherwise progressive increase in creatinine blood levels. Despite its value as a marker of kidney injury, blood creatinine concentrations may not be closely correlated with creatinine clearance in the pig, because in this species some basal tubular creatinine secretion may be present [37]. Moreover, in the context of the significantly higher urine output, the lower blood creatinine levels, and the attenuated tubular TUNEL staining, the significantly higher fractional Na^+ ^excretion probably mirrors the physiologic response to AVP [38] rather than deteriorated tubular function: intravenous AVP increased fractional Na^+ ^elimination both under healthy [39,40] and pathologic conditions [35,41]. Finally, the reduced noradrenaline requirements may have also contributed to the higher fractional Na^+ ^excretion: noradrenaline *per se *was demonstrated to reduce Na^+ ^elimination [42,43].

Several mechanisms may explain the AVP-related less severe organ dysfunction and tissue injury. First, AVP was associated with significantly lower IL-6 levels, that is, an attenuated systemic inflammatory response, which is in good agreement with the anti-inflammatory properties of AVP reported in endotoxic mice [44]. In addition, infusing AVP reduced the amount of exhaled NO, which confirms our own data during terlipressin infusion in endotoxic swine [33], as well as the inhibition of the inducible isoform of the NO synthase in endotoxic rats with biliary cirrhosis [45]. In addition to anti-inflammatory properties of vasopressin *per se*, the lower noradrenaline doses may have attenuated the inflammatory response: catecholamines may mimick [46] and/or enhance [47,48] the inflammatory effects of endotoxin. Second, AVP was affiliated with a smaller rise in the endogenous glucose production rate, while glucose oxidation was identical. Consequently, the percentage of direct, aerobic glucose oxidation as a fraction of endogenous glucose release was significantly increased. Such a switch in fuel utilization to the preferential use of glucose improves the yield of oxidative phosphorylation: the ratio of ATP synthesis to O_2 _consumption is higher for glycolysis than for β-oxidation, because reduced nicotineamide adenine dinucleotide (NADH) as an electron donor provides three coupling sites rather than two only provided by reduced flavine adenine dinucleotide (FADH_2_) [49]. Again, it remains open whether this effect is due to AVP *per se *and/or the reduced catecholamine requirements: Noradrenaline increases endogenous glucose release [50], and Regueria and colleagues showed improved liver mitochondrial function during noradrenaline administration in endotoxic swine [51], whereas other authors emphasized the catecholamine-induced derangement of metabolic efficiency [52].

### Limitations of the study

Mean blood pressure was significantly lower in the control group during the last six hours of the experiment due to the resuscitation protocol imposing a maximum noradrenaline infusion rate at heart rates of 160 beats/min or higher. Hence, any beneficial effect of AVP on organ function and/or damage could be referred to a higher perfusion pressure [53]. We think, however, that the lower blood pressure was unlikely to induce visceral organ ischemia: one control animal only became hypotensive with a mean blood pressure below the range reported to be associated with unchanged parameters of visceral organ perfusion and function in patients with septic shock [54,55]. Moreover, organ blood flow and O_2 _delivery was always well maintained and portal drained viscera O_2 _extraction, hepatic O_2 _uptake, regional venous O_2 _saturation, and lactate/pyruvate ratios were identical.

We used hydroxyethyl-starch for fluid resuscitation, because in swine this colloid caused less pulmonary dysfunction than Ringer's lactate [56] and attenuated capillary leakage [57]. Although we cannot definitely exclude that a hydroxyethyl-starch overload contributed at least in part to the kidney dysfunction [58], this issue most likely did not assume any importance for the difference between the AVP and control animals: both groups received identical colloid resuscitation.

Finally, we investigated young and otherwise healthy pigs during the first 24 hours of sepsis, which precludes any conclusion on the safety of AVP infusion with respect to organ injury during prolonged administration and/or with underlying ischemic heart disease, congestive heart failure, or peripheral vascular disease.

## Conclusions

In our clinically relevant model of fecal peritonitis-induced septic shock, low-dose AVP infusion supplemented with noradrenaline proved to be safe with respect to myocardial and visceral organ function and tissue integrity. Nevertheless, as we observed a reduced dp/dt_max _in young animals without underlying heart disease, the use of AVP should be cautioned in patients with heart failure and/or cardiac ischemia, such as in the recent VASST [27]. It remains to be elucidated whether the attenuated inflammatory response and improved energy metabolism during AVP was due to the treatment *per se *and/or to the reduced noradrenaline requirements needed to achieve the hemodynamic targets.

## Key messages

• Low-dose AVP appears to be safe with respect to myocardial function and heart injury and even attenuates kidney and liver dysfunction and tissue damage during well-resuscitated porcine septic shock.

• An increased aerobic glucose oxidation and reduced hyperlactatemia suggests improved cellular energy metabolism, which coincides with less severe systemic inflammation.

• It remains to be elucidated whether this is due to the treatment *per se *and/or to the decreased exogenous catecholamine requirements.

## Abbreviations

ALAT: alanine aminotransferase; ASAT: asparatate aminotransferase; AVP: arginine vasopressin; CO_2_: carbon dioxide; dp/dt_max_: maximal systolic contraction; dp/dt_min_: maximal diastolic relaxation; FADH_2_: reduced flavine adenine dinucleotide; FiO_2_: fraction of inspired oxygen; H&E: hematoxylin and eosin; I/E: inspiratory-to-expiratory; IL-6: interleukin-6; NADH: reduced nicotineamide adenine dinucleotide; NO_2_^-^+NO_3_^-^: nitrate+nitrite; O_2_: oxygen; PaO_2_: partial pressure of arterial oxygen; PaCO_2_: partial pressure of arterial carbon dioxide; PEEP: positive end-expiratory pressure; τ: diastolic relaxation time constant; TNFα: tumor necrosis factor-α; TUNEL: terminal deoxynucleotidyltransferase-mediated nick-end labeling assay; VASST: vasopressin and septic shock trial.

## Competing interests

RL is a full-time salaried employee of Ferring Research Institute Inc., San Diego, CA, USA. PA, PR, and EC received a research grant from Ferring Research Institute Inc., San Diego, CA, USA. PR and PA received consultant fees from Ferring Pharmaceutical A/S, København, Denmark, for help with designing preclinical experiments. The other authors declare that they have no competing interests.

## Authors' contributions

PA, RL, PR, and EC played a pivotal role in planning and designing the experimental protocol. FS, MG, and FP carried out the anesthesia, surgical instrumentation as well as the on-line data collection. RG, BH, and MG were responsible for the data analysis. AS and PM provided the histomorphology and immunohistochemistry findings and the analysis of these data. JV and UW were responsible for the isotope data acquisition, analysis, and interpretation. MG, PR, and BH wrote the manuscript.
